# Wnt signaling induces differentiation of progenitor cells in organotypic keratinocyte cultures

**DOI:** 10.1186/1471-213X-7-9

**Published:** 2007-02-17

**Authors:** Marni A Slavik, B Lynn Allen-Hoffmann, Bob Y Liu, Caroline M Alexander

**Affiliations:** 1 Department of Pathology, University of Wisconsin Medical School-Madison, Madison, Wisconsin, USA; 2 McArdle Lab for Cancer Research, University of Wisconsin Medical School-Madison, Madison, Wisconsin, USA; 3 Saint Michael's College, Department of Biology, One Winooski Park, Colchester, VT 05439, USA; 4 Dept. Pathology, HSW 451, University of California, San Francisco, 513 Parnassus Ave, San Francisco, CA 94143-0511, USA

## Abstract

**Background:**

Interfollicular skin develops normally only when the activity of the progenitor cells in the basal layer is counterbalanced by the exit of cells into the suprabasal layers, where they differentiate and cornify to establish barrier function. Distinct stem and progenitor compartments have been demonstrated in hair follicles and sebaceous glands, but there are few data to describe the control of interfollicular progenitor cell activity. Wnt signaling has been shown to be an important growth-inducer of stem cell compartments in skin and many other tissues.

**Results:**

Here, we test the effect of ectopic Wnt1 expression on the behavior of interfollicular progenitor cells in an organotypic culture model, and find that Wnt1 signaling inhibits their growth and promotes terminal differentiation.

**Conclusion:**

These results are consistent with the phenotypes reported for transgenic mice engineered to have gain or loss of function of Wnt signaling in skin, which would recommend our culture model as an accurate one for molecular analysis. Since it is known that canonical ligands are expressed in skin, it is likely that this pathway normally regulates the balance of growth and differentiation, and suggests it could be important to pathogenesis.

## Background

The homeostatic architecture of the skin is maintained by a tightly regulated balance between proliferation and differentiation, which occurs continuously as the skin self-renews. In adult skin, growth potential is focused into a minor subpopulation of stem and progenitor cells, some located in hair follicles and others in sebaceous glands [1,2]. These have been shown to be particularly important to wound healing [3] and also to the regenerative cycle of the hair follicle [4]. The growth potential of interfollicular keratinocytes resides in the progenitor population within the basal layer [5]. These cells are distributed into developmental fields, which divide laterally to become rapidly expanding transit amplifying cells, and differentiate after detachment from the basal lamina, migrating up into the stratified suprabasal layers of the skin [6,7].

Recently, some of the soluble factors that control progenitor cell proliferation and differentiation have been identified. Ectopic activation of the Wnt signaling pathway, in particular, has been shown to promote progenitor dysfunction in skin, as it does for other lineages [1,8,9]. Thus, transgenic mice engineered to have gain of function of Wnt signaling in basal keratinocytes, showed *de novo *hair follicle morphogenesis, follicular hyperplasia and tumors [9]. The canonical Wnt signaling pathway (mediated by β-catenin) is indeed highly oncogenic for a number of mammalian lineages, and there is gathering evidence that the underlying mechanism is the misregulation of stem cell compartments. While the role of Wnt signaling in follicular keratinocytes has been well established, its function in interfollicular keratinocyte regulation is unknown.

Wnt ligand expression (Wnt 4, 5a, 10b and 11) has been observed in interfollicular skin [3] (and MS and BLA-H, unpublished), together with a number of the other components of the Wnt signaling pathway, implying that this pathway may be involved in normal morphogenesis. Here, we tested the effect of mis-expression of the canonical Wnt ligand, Wnt1, on development of an organotypic culture model of interfollicular skin.

Our interfollicular organotypic culture model is based on a cell line that arose spontaneously from human foreskin keratinocytes (Normal Immortalized Keratinocytes; NIKs). These cells replicate all aspects of differentiation *in vivo*, and are used therapeutically for grafting to human patients. They differentiate normally in culture as well, and resemble the pattern typical of primary keratinocyte cultures. NIKs are non-tumorigenic and have a stable, near-diploid karyotype [10]. They grow in monolayer culture as undifferentiated, highly proliferative cells, but when transferred to organotypic culture on collagen gels containing human fibroblasts, they stratify, closely resembling their epidermal counterpart *in vivo*. Every molecular marker so far tested shows normal expression.

Our results were surprising. Unlike the proliferative response of follicular stem cells to ectopic Wnt signaling in vivo, interfollicular progenitor cells were induced to exit from the proliferative compartment and undergo terminal differentiation, suggesting that the response to Wnt signaling is highly dependent upon the keratinocyte cell context.

## Results

### Cultured human keratinocytes transduced with Wnt1 show reduced proliferation and an increase in cell size

**Figure 1:**
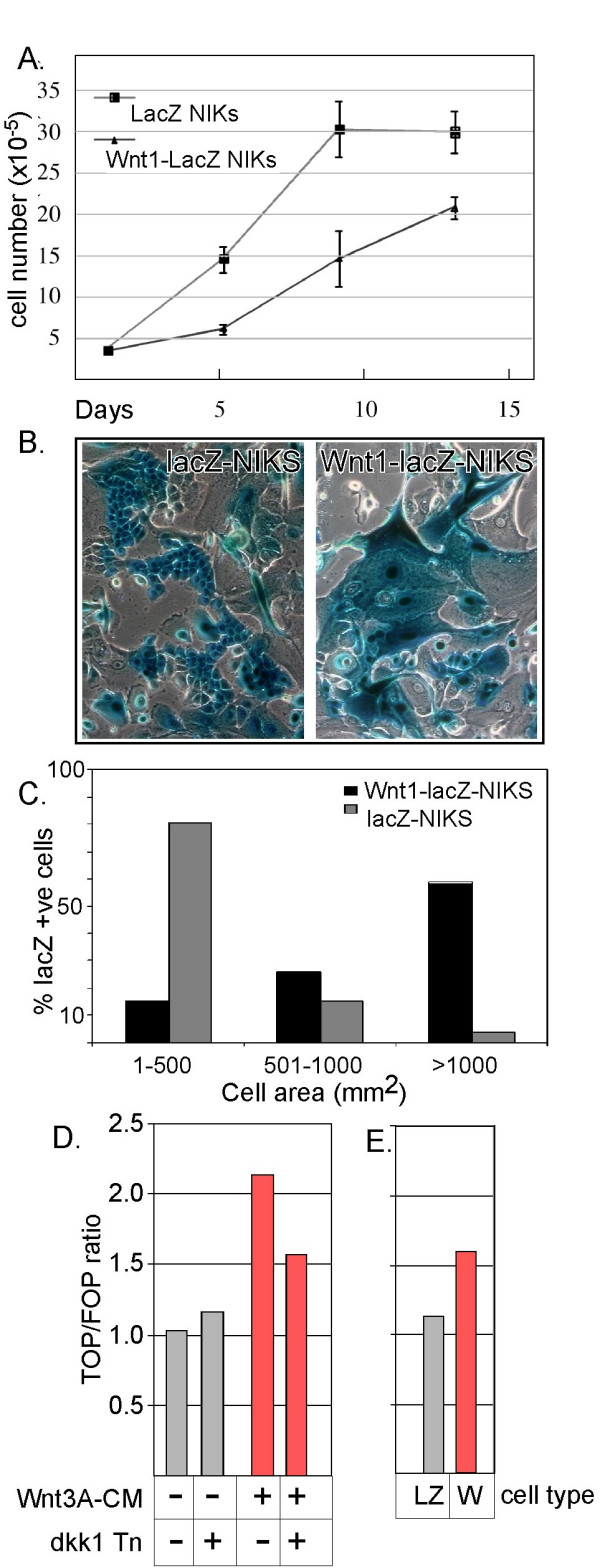
***Wnt1 expression slows growth and increases cell size in monolayer cultures of NIKs cells***. The growth of NIKs cells transduced either with a control retrovirus (lacZ) or a Wnt1 expression vector (Wnt1-IRES-lacZ) was compared (A: total cell counts). When cultures were stained for lacZ expression (B), the number of large and very large cells expressing Wnt1 was greatly increased (C). Canonical Wnt signaling was measured as fold induction of a consensus TCF-βcatenin reporter (TOP-FLASH) with respect to FOP-FLASH (a scrambled consensus reporter, all normalized for transfection efficiency as previously described). The TOP/FOP ratio was increased in NIKs cells by 2× when cells were incubated with soluble (crude) Wnt3A for 22 hours, and reduced if the cells were transfected with the canonical Wnt inhibitor, dkk1, prior to Wnt3A incubation (D). Cell strains stably transduced with retroviral constructs (LZ, lacZ control; W, Wnt1-lacZ test vector) were transfected with either FOP-FLASH or TOP-FLASH reporters (E). The Wnt1-expressing culture showed a significant increase in Wnt-dependent transactivation (n = 3), though less than the naïve cultures treated with soluble Wnt3A (probably because of negative feedback).

Monolayer cultures of NIKS keratinocytes were transduced with either a *Wnt1*-IRES-*lacZ *retrovirus or a control virus, IRES-*lacZ*. Approximately 85% of NIKS cells were infected (determined by β-galactosidase staining). Both *Wnt1*-IRES-*LacZ *and IRES-*LacZ *transduced NIKS cell lines were expanded for eight passages. Prior to each experiment, the percentage of *lacZ*-expressing cells was re-assessed and found to be approximately constant. We measured the effect of Wnt1 expression on the proliferative capacity of keratinocyte monolayers, and found that it was reduced by 50% (Fig. 1A). In monolayer culture, keratinocytes undergo an attenuated differentiation program, generating a heterogeneous culture population that contains small, actively dividing cells, together with a proportion of large, terminally differentiated cells. In cultures of keratinocytes transduced with *Wnt1*-IRES-*LacZ*, there were many more large cells (Fig. 1B). We quantified the area of transduced cells, and observed that there was a 20 fold increase in very large cells (>1000 μm^2^; Fig. 1C).

Specific induction of TCF-βcatenin-dependent transactivation in response to ectopic Wnt signaling was measured using the canonical TOP-FLASH reporter. (The fold induction of reporter expression is expressed as a ratio with respect to the control scrambled reporter, FOP-FLASH). To evaluate the induction of TOP-FLASH expression in NIKs cells, cultures were treated with soluble Wnt3A (a canonical Wnt ligand that shares all the properties of insoluble Wnt1 characterized so far; [11]). These cultures showed a 2× increase in Wnt reporter expression. This was reduced by co-expression of the canonical Wnt pathway inhibitor, dkk1 (Fig. 1D). Transactivation of TOP-FLASH was increased in cell strains constitutively expressing Wnt-1 from the viral expression vector (W) compared to control virus (LZ) (Fig. 1E).

Thus Wnt1 expression results in decreased cell proliferation and increased cell size, and suggests that Wnt signaling affects the balance between proliferation and differentiation. To test this proposal more rigorously, we transferred the cells to organotypic culture.

### Interfollicular epidermis generated from Wnt1-IRES-LacZ keratinocyte populations shows precocious thickening of the stratum corneum and depletion of nucleated cells from the basal layer

**Figure 2:**
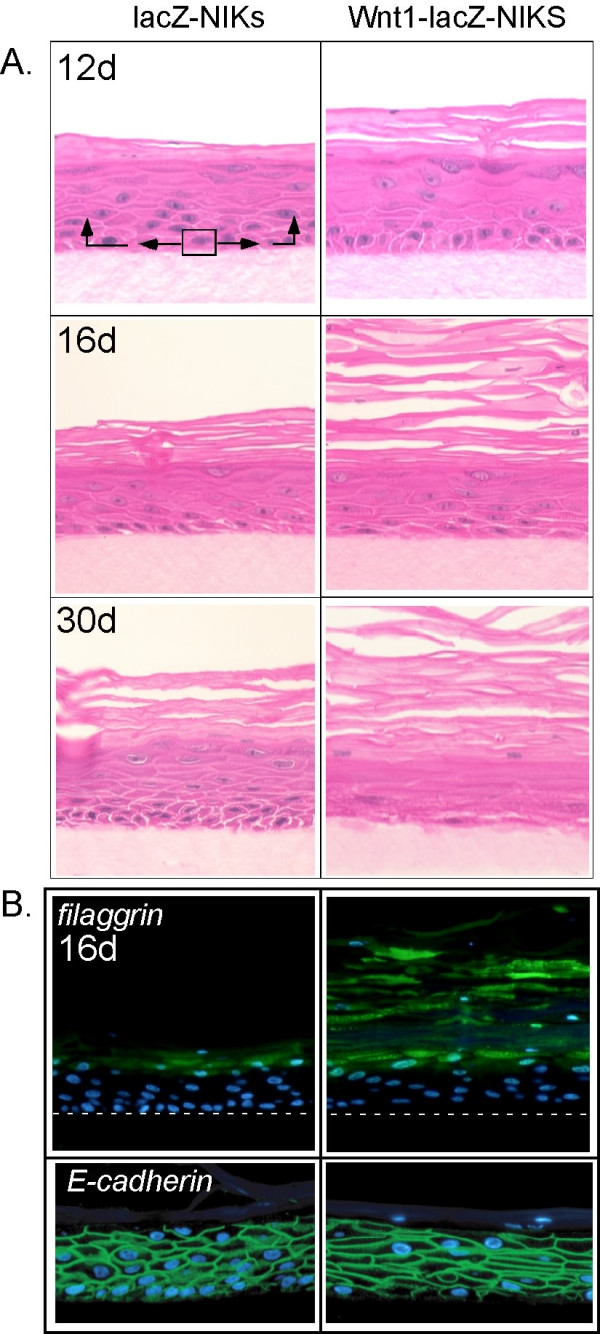
***Wnt1 expression accelerates differentiation of organotypic cultures***. NIK cells were transferred to culture conditions that promote epidermal stratification and development. Samples were taken at the timepoints indicated (12, 16 and 30 days), embedded, sectioned and stained with H&E (A) to reveal their z-axis morphology. The arrows indicate the pattern of growth typical of these cultures, relying on progenitor cells to feed the basal transit amplifying population that differentiate upwards into the stratified layers. Similar samples were processed for filaggrin-staining (B), and cell outlines were visualized with an antibody to E-cadherin.

Wnt1-IRES-*LacZ *and control IRES-*LacZ *NIKs cells were seeded at equal cell density onto dermal equivalents. NIKs cultures proliferate to form a continuous sheet of cells, and at confluence, cells begin to stratify as they undergo differentiation. After 12 days, control cultures had organized into a stratified epidermis, and cornification proceeded for several weeks thereafter). Epidermal tissue generated from Wnt 1-IRES-*LacZ *keratinocyte populations exhibited a thickened stratum corneum (hyperkeratosis) relative to control IRES-LacZ keratinocytes at all time points (Fig. 2). To test whether the hyperkeratotic layers were expressing differentiated molecular markers, we confirmed that the suprabasal layers of differentiating cultures of Wnt1-lacZ-NIKs cells were filaggrin-positive (Fig. 2; fillagrin is a late stage marker of interfollicular differentiation expressed specifically in the granular layer and stratum corneum [12]).

The basal layer of the Wnt 1-IRES-*LacZ *epidermis became progressively more orthokeratotic or enucleated over the course of 30 days. Cells in Wnt-1 expressing organotypic culture showed an elongated, flat morphology compared to normal cells (Fig. 2; cells are outlined by staining for E-cadherin).

**Figure 3:**
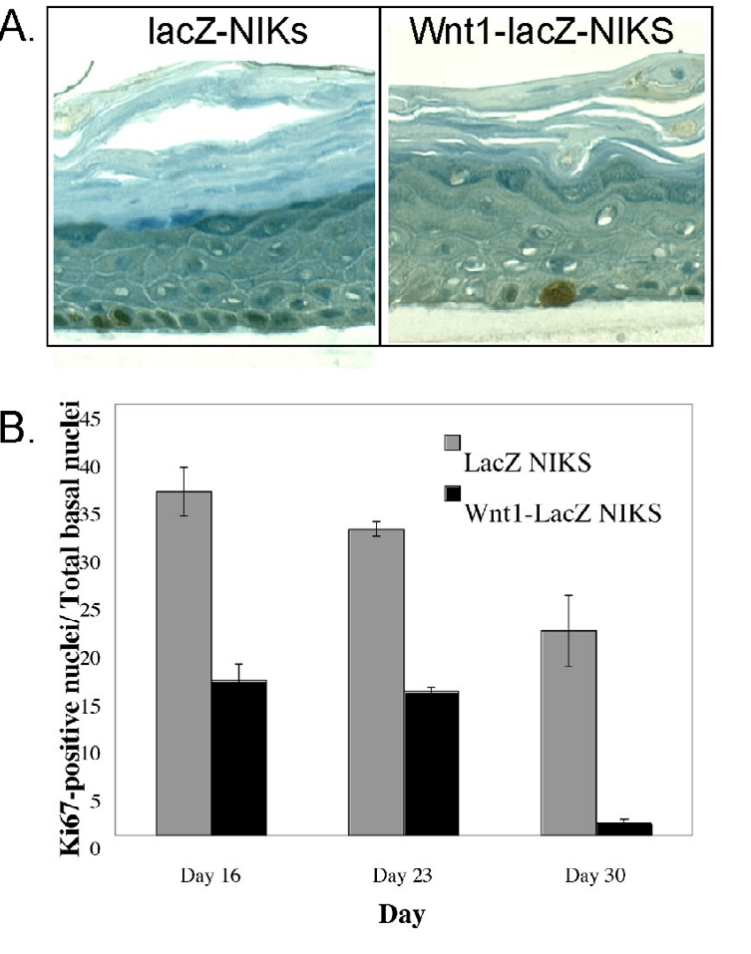
***The mitotic activity of the Wnt1-expressing basal layers is reduced***. Samples described in the legend to Fig. 2 were immunostained for Ki67 (A), and the number of Ki67-positive cells per basal cell nucleus quantified (B).

We determined the proportion of dividing basal cells in Wnt1-expressing cultures by immunostaining for the M phase cell cycle marker Ki67. Over the course of 30 days, control cultures show a decrease in Ki67-positive nuclei (by approximately 30%; Fig. 3). In Wnt1-expressing cultures, the mitotic index was reduced by 50% on day 16, and this was reduced to almost zero after 30 days (Fig. 3A). Thus, after 1 month in culture, there was very little growth activity in cultures expressing Wnt1.

### Wnt1 expressing-cells are progressively excluded from the dividing basal layer

**Figure 4:**
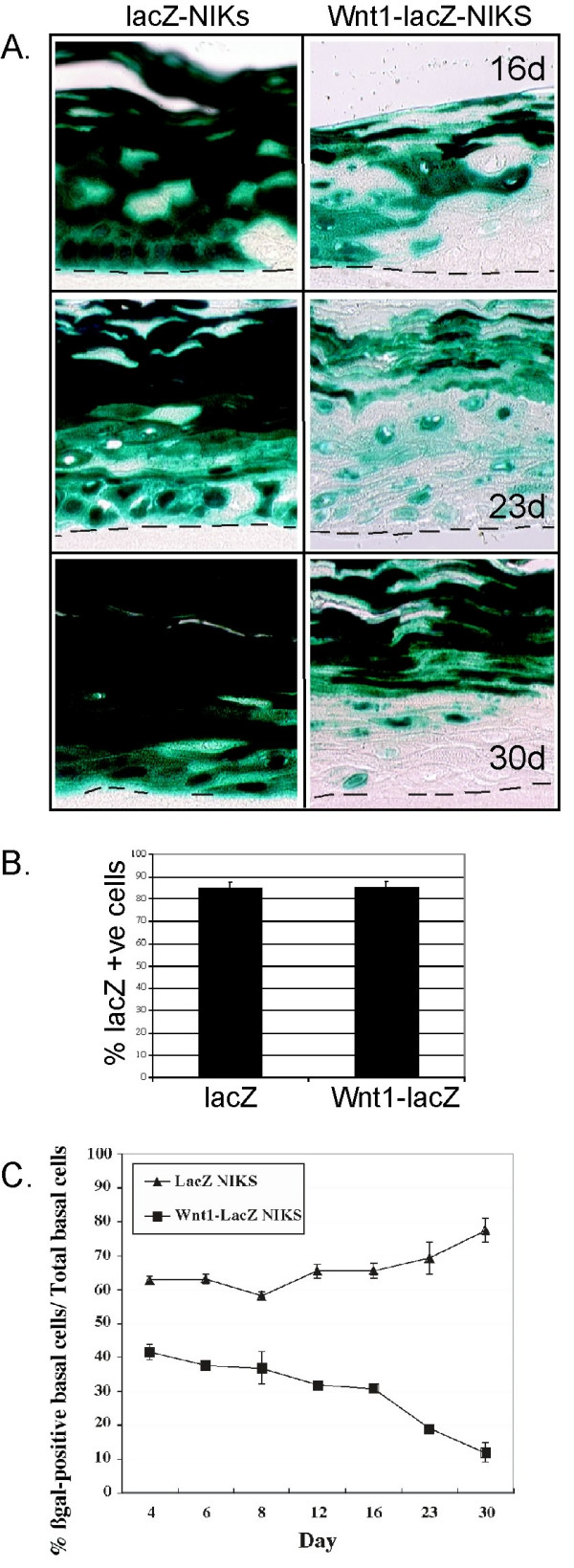
***Wnt-expressing keratinocytes are progressively excluded from the basal replicative layer***. Samples described in the legend to Fig. 2 were stained for *lacZ *expression to illustrate the distribution of Wnt1- (or control) expressing cells. Panel B) shows the proportion of cells that expressed *lacZ *for each cell strain at plating. The exclusion of *lacZ-*positive basal cells was quantified over time (C).

We assessed the distribution of *lacZ-*expressing cells in organotypic cultures from control IRES-*lacZ *and Wnt1-IRES-*lacZ *cultures. Whereas the control cultures maintained a constant majority of *lacZ*-positive cells during the culture period (Fig. 4), the Wnt1-expressing cultures showed a 75% reduction of *lacZ*-positive cells in the basal layer over the course of 30 days (Fig. 4). This loss of *lacZ *expression spread upwards into the suprabasal layers as the culture matured, suggesting that Wnt1-expressing basal cells were eliminated from the tissue over time.

### Wnt-induced growth inhibition and stratification is reversed by Dkk1, a canonical Wnt signaling inhibitor

**Figure 5:**
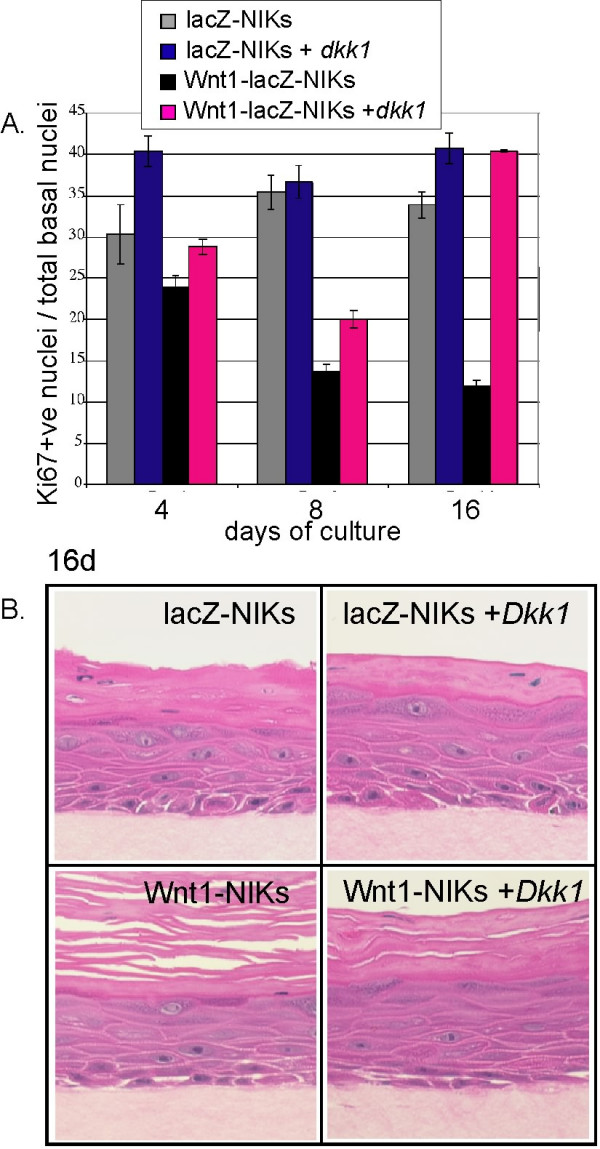
***The canonical Wnt signaling inhibitor, Dkk1, rescues Wnt-induced hyperkeratosis***. NIK cultures were incubated with or without soluble *dkk1 *for 30 days, and the mitotic index of the basal layer of the organotypic cultures was assayed (A). The same cultures were stained with H&E, and cut on the z-axis to evaluate their differentiation (B).

The canonical, β-catenin-TCF Wnt transactivation pathway is specifically inhibited by the extracellular, soluble inhibitor, Dkk1 [13-17]. We added Dkk1 to keratinocyte cultures in order to test whether the canonical Wnt pathway was responsible for the observed growth inhibition and precocious differentiation. Morphologically, the hyperkeratosis was rescued in large part by the addition of soluble, recombinant Dkk1 to the cultures (Fig. 5B), and this was confirmed by counting the proportion of Ki67-positive basal nuclei (Fig. 5A). Thus, we implicate the canonical activity of Wnt1 as an important regulator of the choice of basal cells to proliferate or differentiate.

## Discussion and conclusion

Canonical Wnt ligands are expressed in normal human interfollicular skin [3] (and MS and BLA-H, unpublished), suggesting that this pathway may be used normally to regulate the maturation of the keratinocyte lineage during growth and differentiation. It is known that gain- or loss-of function of this pathway has profound effects on growth and differentiation for stem and progenitor cells in hair follicles and sebaceous glands in transgenic mice. Thus, in [K14-ΔNβ catenin] transgenic mice (expressing a non-degradable β-catenin Wnt signaling effector in basal keratinocytes) there was an increase in folliculogenesis, and ectopic proliferation of cells in stem cell compartments, leading eventually to tumor development [9,18]. Expression of ΔN-Lef1 induced cysts and sebaceous tumors [19]. Subtle manipulations in vivo have revealed that Wnt signaling reduces the threshold for activation of follicular stem cell division [20]. These authors propose that the stem cell niche has a powerful inhibitory function, maintaining stem cell quiescence, and Wnt signaling overcomes this signal to initiate tissue growth.

Epidermal stem cells (though usually separate pools) are able to differentiate along any of the epidermal lineages (follicular, sebocyte and interfollicular), given the correct microenvironment [1,21]. Misregulation of one stem cell compartment tends to generate complex phenotypes in the other lineages. Since there is no unambiguous way to dissociate the activity of the follicular and interfollicular compartments in transgenic mice [22], we have used an organotypic culture model of interfollicular skin to isolate the effects of Wnt signaling upon the interfollicular keratinocytes.

We have shown that the ectopic expression of the canonical Wnt signaling pathway shifts the balance of division and differentiation for interfollicular progenitor cells away from cell division towards precocious differentiation. If our culture model were accurate, we would predict that inhibition of Wnt signaling in basal cells of transgenic mice should increase growth and reduce differentiation. Indeed, hyperproliferation of interfollicular skin was observed in mice expressing the dominant negative transactivation inhibitor, ΔN-Lef1 [19]. Similarly, transgenic mice expressing Tcf3 (described as a transactivation repressor) showed decreased expression of filaggrin and loricrin within the interfollicular epidermis [18]. We propose therefore that this culture model can be used to accurately model of interfollicular development.

The outcomes of Wnt signaling are known to be highly context-dependent. Here we show that although Wnt signaling has previously been shown to promote proliferation of follicular stem cells, it induces the differentiation of interfollicular progenitors. Wnt signaling is a classic morphogenic pathway known to regulate cell fate choices and differentiation during developmental processes [23], for example those associated with imaginal disc formation. Thus, in ommatidial development, Wnt signaling is used early during specification, and later to promote differentiation and apoptosis of peripheral retinal cells [24]. Within mammalian lineages, Wnt signaling has been shown to be key to opposite cellular growth/differentiation choices [25]. Wnt signaling promotes hematopoietic stem cell proliferation, and is used again later in the lineage to promote T cell differentiation [26]. Similarly, Wnt signaling promotes intestinal crypt stem cell division and accumulation, and later in the lineage induces maturation of Paneth cells [27-29]. Gain of function of Wnt signaling in neural crest cells generates sensory neurons at the expense of all other lineages, re-specifying cell fate and promoting differentiation [30].

Organotypic keratinocyte culture establishes a balance between basal cell renewal and differentiation resulting in the continued accumulation of fully differentiated squames in the stratum corneum over time. Upon transplantation of fully stratified organotypic cultures to athymic mice, the balance between basal cell renewal and differentiation becomes further normalized, supporting long term renewal of the interfollicular epidermis [31]. These early studies coupled with our findings suggest that basal progenitor compartment is maintained in organotypic keratinocyte cultures. Applying the paradigm derived from other differentiating lineages, we suggest that ectopic Wnt signaling accelerates the exit of cells from the basal progenitor compartment, rapidly reducing stem cell activity. We conclude that this organotypic culture model could be used to screen for molecular candidates that regulate the interfollicular stem/progenitors cell niche, a niche that maintains the renewal potential of skin.

## Methods

### Culture of human keratinocytes

The spontaneously immortalized human keratinocyte cell line NIKS was grown either in monolayer culture on 3T3-fibroblasts, or in organotypic culture in keratinocyte growth media on a simulated dermal raft [10] with the following modification: The dermal component was provided by Stratatech Corp. (Madison, WI) (formed by mixing normal human neonatal fibroblasts with type 1 collagen on hyaluronic acid (HA) membranes in Millicell cell culture plate inserts (10 mm diameter). Cells were plated at 3.5 × 10^5 ^cells in 150 μls of Stratalife™ media 1 (Stratatech Corp) per dermis, and the outer well was flooded with media 1. Two days post-plating, cultures were fed with Stratalife™ media 2, and day 4 post-plating were re-fed with Stratalife™ media 3, and media changed every other day thereafter.

### Ectopic Wnt expression

The construction of retroviral expression vectors expressing Wnt1 and Dkk1 were described in Liu et al (2003) [32]. To make virus, 293 cells were transfected with 3 plasmids simultaneously, pcMMP Wnt1-IRES-lacZ, pMMP-VSV-G, and pMMP gag-pol (4 × 10^6 ^cells transfected with 10 μgs, 5 μgs and 5 μgs plasmids respectively, and 10 μls of Lipofectamine 2000). Virus was harvested 48 and 72 hours post-transfection, filtered, concentrated by ultracentrifugation and stored at -80°C. Viral titer was determined by measuring the MOI after infection of 293 cells (cells were infected in 4 mg/ml polybrene for 1 hour/4°C). NIKs were transduced with Wnt1-lacZ, or lacZ viruses, at MOI 2.5. Where indicated, recombinant human Dkk1 (R&D, Minneapolis, MN) was added to organotypic culture media at a final concentration of 0.1 μg/ml, or cultures were transfected with a viral pcMMP Dkk1IRES-lacZ expression construct (0.1 μgs).

Assays of transactivation of the Wnt reporter, TOP-FLASH (together with the scrambled control construct, FOP-FLASH) were as described in Liu et al (2003) [32]. Briefly, samples were measured in triplicate, and readings normalized for transfection efficiency using the co-transfected Renilla luciferase standard. Results were expressed as fold induction of TOP-FLASH expressed as a ratio with respect to FOP-FLASH. Production of Wnt3A conditioned medium was described by Liu et al (2003) [32].

### Immunohistochemistry and image analysis

Organotypic cultures were fixed for 120 mins in 1% paraformaldehyde; half of each tissue was cryopreserved (equilibrated in 20% sucrose in PBS, 4°C overnight, and embedded in OCT (Tissue Tek), and the other half post-fixed in 10% buffered formalin, followed by paraffin-embedding. For detection of β-galactosidase (lacZ) activity in organotypic cultures, 5 μm frozen sections were fixed in acetone (5 min, -20°C), air dried, post-fixed in 2% formaldehyde/0.2% glutaraldehyde (5 min, 4°C) and incubated with X-gal staining solution (4 mM potassium ferricyanide, 4 mM potassium ferrocyanide, 2 mM magnesium chloride, 1 mg/ml X-Gal at 37°C for upto 72 hr). For immunofluorescent analysis, 5 μm sections of paraffin-embedded organotypic cultures were stained with antibodies against filaggrin (NeoMarkers, Fremont, CA), E-cadherin (Transduction Labs, KY) and Ki67 (Novo Castra, Newcastle, UK; all primary antibodies used at 2 – 5 μg/ml). (For filaggrin staining, sections were microwaved in 10 mM sodium citrate pH 6.0 to promote antigen exposure). Sections were blocked with 10% goat serum (Sigma, St. Louis, MO) in PBS, and incubated in primary antibodies for 30–60 minutes at room temperature, washed, incubated with secondary antibodies (Alexa 488-conjugated goat anti-mouse IgG (Molecular Probes, Eugene, OR) or biotinylated Universal antibody, VectaStain Elite kit (Vector Laboratories, Burlingame, CA, used as recommended). The immunohistochemical stain for Ki67 was developed using the Vectastain Elite ABC reagent (Vector Laboratories, Burlingame, CA). Immunofluorescent stains were counterstained with 5 μg/ml Hoechst 33258, and immunohistochemical stains with Harris Hematoxylin for 50 sec. To assess the average area of individual cells in monolayer culture, NIH Image software [33] was used to assess images captured using an inverted microscope.

## Abbreviations

NIKS: Near-diploid Immortal Keratinocytes

## Authors' contributions

MS complete this work as part of her PhD thesis; B.L.A-H co-mentored MS and provided resources; BL developed the materials and methods associated with testing gain and loss of function for Wnt signaling in epithelial cells, and advised MS on experimental design; CMA was co-mentor for MS, advising on strategy and interpretation, and wrote and submitted the manuscript. All authors read and approved the final manuscript.
